# Preparation of Zwitterionic Sulfobetaines and Study of Their Thermal Properties and Nanostructured Self-Assembling Features

**DOI:** 10.3390/nano15010058

**Published:** 2025-01-02

**Authors:** Yenglik Amrenova, Arshyn Zhengis, Arailym Yergesheva, Munziya Abutalip, Nurxat Nuraje

**Affiliations:** 1 Renewable Energy Laboratory, National Laboratory Astana, Nazarbayev University, Astana 010000, Kazakhstan; yenglik.amrenova@nu.edu.kz (Y.A.); arshyn.zhengis@nu.edu.kz (A.Z.); arailym.yergesheva@nu.edu.kz (A.Y.); 2 Department of Chemical and Biochemical Engineering, Satbayev University, Almaty 050013, Kazakhstan; 3 School of Chemistry and Chemical Technology, Al-Farabi Kazakh National University, Almaty 050040, Kazakhstan

**Keywords:** polyzwitterions, self-assembly, thermal properties, glass transition temperature

## Abstract

Zwitterionic polymers have garnered significant attention for their distinctive properties, such as biocompatibility, antifouling capabilities, and resistance to protein adsorption, making them promising candidates for a wide range of applications, including drug delivery, oil production inhibitors, and water purification membranes. This study reports the synthesis and characterization of zwitterionic monomers and polymers through the modification of linear, vinyl, and aromatic heterocyclic functional groups via reaction with 1,3-propanesultone. Four zwitterionic polymers with varying molecular structures—ranging from linear to five and six membered ring systems—were synthesized: poly(sulfobetaine methacrylamide) (pSBMAm), poly(sulfobetaine-1-vinylimidazole) (pSB1VI), poly(sulfobetaine-2-vinylpyridine) (pSB2VP), and poly(sulfobetaine-4-vinylpyridine) (pSB4VP). Their molecular weights, thermal behavior, and self-assembly properties were analyzed using gel permeation chromatography (GPC), thermogravimetric analysis (TGA), differential scanning calorimetry (DSC), transmission electron microscopy (TEM), and zeta potential measurements. The glass transition temperatures (Tg) ranged from 276.52 °C for pSBMAm to 313.69 °C for pSB4VP, while decomposition temperatures exhibited a similar trend, with pSBMAm degrading at 301.03 °C and pSB4VP at 387.14 °C. The polymers’ self-assembly behavior was strongly dependent on pH and their surface charge, particularly under varying pH conditions: spherical micelles were observed at neutral pH, while fractal aggregates formed at basic pH. These results demonstrate that precise modifications of the chemical structure, specifically in the linear, imidazole, and pyridine moieties, enable fine control over the thermal properties and self-assembly behavior of polyzwitterions. Such insights are essential for tailoring polymer properties for targeted applications in filtration membranes, drug delivery systems, and solid polymer electrolytes, where thermal stability and self-assembly play crucial roles.

## 1. Introduction

Among various compounds, zwitterionic polymers are distinguished by their unique structure, incorporating both cationic and anionic groups while maintaining an overall neutral charge. The first zwitterionic material, phosphorylcholine (PC) head groups, has been in use since the 1990s. However, PC-based monomers, such as 2-methacryloyloxyethyl phosphorylcholine (MPC), are prone to moisture absorption, have low yields, and are challenging to synthesize, which contributes to high production costs. Consequently, researchers have been motivated to find more effective alternatives [1,2]. Over time, zwitterionic polymers like sulfobetaine (SB) and carboxybetaine (CB) have emerged as promising substitutes for PC materials. SB-based polymers are particularly advantageous due to their biomimetic properties, biocompatibility, noncytotoxicity, and ease of synthesis. Additionally, the carboxylic acid groups in carboxybetaine methacrylate (CBMA) can be easily converted into various functional groups [3,4,5].

Polyzwitterions (PZIs) are valuable across a range of applications due to their distinctive structure. Their high biocompatibility stems from zwitterionic side groups that mimic phospholipid head groups, making them suitable for wound dressings and drug delivery systems [6,7,8,9,10]. In filtration applications, the zwitterionic structure confers antifouling properties to membranes. Moreover, PZIs are adept at dissolving salts, positioning them as ideal candidates for solid polymer electrolytes.

Research by Yu et al. highlighted the role of zwitterionic materials in aqueous zinc-based energy storage systems, demonstrating their capacity to enhance electrolyte stability and ion conductivity while mitigating challenges such as zinc dendrite formation [11]. Similarly, Yuan et al. explored zwitterionic materials in biological applications, particularly in adeno-associated virus (AAV)-mediated gene therapy. Their findings indicated that an immunosuppressive phosphoserine-containing zwitterionic peptide modified AAV vectors, maintaining transduction efficiency while significantly reducing immune responses, which facilitated successful re-administration of AAV vectors [11].

Zwitterionic polymers are highly valued for their ability to enhance colloidal stability and provide stealth properties for biomedical applications. The zwitterionic groups create a hydrated surface layer that prevents aggregation and non-specific adsorption, making them ideal for colloidal formulations, such as nanoparticles. Additionally, their “stealth” behavior, by mimicking the natural glycocalyx on cell membranes, reduces immune detection, improving the circulation time and bioavailability of therapeutic agents. These properties are particularly beneficial for drug delivery and gene therapy, where stability and minimal immune responses are crucial [12].

Zwitterionic polymers exhibit unique self-assembly behaviors due to the presence of both positive and negative ionizable groups within the same repeat unit (polybetaine) or in alternating repeating units (polyampholyte) [13]. These groups generate diverse interactions depending on pH, ionic strength, and thermal conditions. Unlike polyelectrolytes, which tend to precipitate at high salt concentrations, polybetaines expand in response to salt addition due to the screening of electrostatic attractive forces. Recently, hydrophobically modified polybetaines have attracted considerable interest for their unique properties [14,15,16,17].

PZIs possess zwitterionic side groups that engage in dipole–dipole interactions both within the same polymer chain (intrachain) and between adjacent chains (interchain). These interactions contribute to the formation of amorphous polymers lacking long-range molecular order while also providing physical crosslinks that enhance mechanical durability. This characteristic makes polybetaines particularly suitable for hydrogel applications, as these physical crosslinks offer substantial stability, leading to polyzwitterions with high glass transition temperatures (Tg). Notably, many polyzwitterions do not exhibit glass transition relaxation prior to thermal degradation when subjected to traditional scanning calorimetry rates. Recent advancements in fast scanning calorimetry have allowed researchers to accurately determine the Tg of various new polyzwitterions [18,19].

Investigations into the solution characteristics of polyzwitterions suggest that the formation of physical crosslinks is thermally reversible. Hydrogel systems made from polyzwitterions show potential for reversible thermal properties [20,21,22,23]. However, comprehensive studies on the thermal characteristics of dry polyzwitterions remain limited. Notably, Clark et al. employed temperature-modulated differential scanning calorimetry (TMDSC) to explore the temperature-dependent specific heat capacity of polyzwitterions, providing valuable insights into their thermal behavior.

In this study, we employed a traditional synthesis method for zwitterionic polymers due to its established reliability and effectiveness in producing materials with desirable properties. Although novel synthesis approaches have emerged, our chosen methodology is straightforward and cost-effective, facilitating the incorporation of functional groups essential for tailoring polymers to specific applications. This synthesis technique is particularly well suited for industrial and biomedical applications, where scalability, efficiency, and control over polymer properties are critical.

This work distinguishes itself from recent literature by not only focusing on the synthesis of novel PZIs but also providing a comprehensive analysis of their thermal and self-assembly properties. Previous studies have highlighted the potential of zwitterionic polymers like SB and CB; however, the influence of various chemical modifications on their thermal behavior—especially the glass transition temperature (Tg) and self-assembly capabilities—has not been thoroughly investigated [24,25,26].

We conducted an extensive study on the thermal and self-assembly characteristics of PZIs utilizing TGA, DSC, and TEM. TGA was employed to quantify the amount of chemically bound water in the PZIs and identify the appropriate temperature range for heat capacity measurements. DSC was used to determine Tg and analyze the thermal behavior of these systems. The self-assembly behavior of the PZIs was investigated via TEM at different pH levels, revealing distinct morphological changes and aggregation patterns influenced by the pH environment.

**Figure 1:**
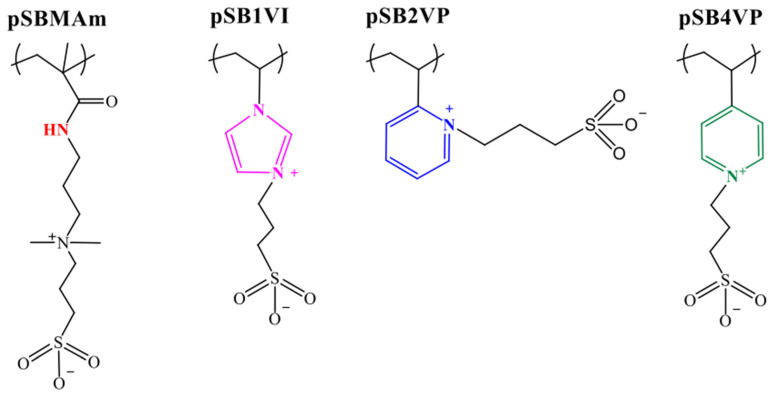
Schematic illustration of zwitterionic polymers investigated in this study.

All polymers studied consist of the same sulfobetaine anion, and we systematically modified the composition of the polymers from the original polyzwitterion, pSBMAm, which features a methacrylamide group connecting the side group to the backbone [27,28,29,30]. The latest PZIs, namely pSB1VI, pSB2VP, and pSB4VP, exhibit significant structural diversity, varying in imidazole and pyridine rings as well as the positions of these rings. In these PZIs, the sulfobetaine group is linked to the polymer backbone through an imidazole or pyridine ring [31,32,33]. Variations in chemical structure can significantly influence the thermal properties of typical polymers, impacting both backbone flexibility and side group bulk. The PZIs illustrated in Figure 1 showcase chemical differences that lead to the presence of larger side groups (pSBMAm, pSB1VI, pSB2VP, and pSB4VP) as well as increased flexibility in the backbones (pSB1VI, pSB2VP, and pSB4VP), enabling a comprehensive exploration of the impact of chemical structure on PZIs [34,35,36].

## 2. Materials and Methods

### 2.1. Materials

*N*,*N*-dimethylaminopropyl methacrylamide (DMAPMA), 2-vinylpyridine (2VP), 4-vinylpyridine (4VP), 1-vinylimidazole (1VI), 1,3-propanesultone (1,3-PS), acetone, acetonitrile, diethyl ether, ethanol, hexane, methanol, azobisisobutyronitrile (AIBN), ammonium persulfate (APS), sodium chloride (NaCI), dimethyl sulfoxide (DMSO), and monomethyl ether of hydroquinone (MEHQ) were obtained from Sigma Aldrich (Darmstadt, DA, Germany) and used as received without purification. Deionized (DI) water with a conductivity of 18.2 MΩ was utilized in all experiments.

### 2.2. Synthesis of Zwitterionic Monomers and Amphiphilic Zwitterionic Polymers

#### 2.2.1. Preparation of Zwitterionic Monomers

**Scheme 1:**
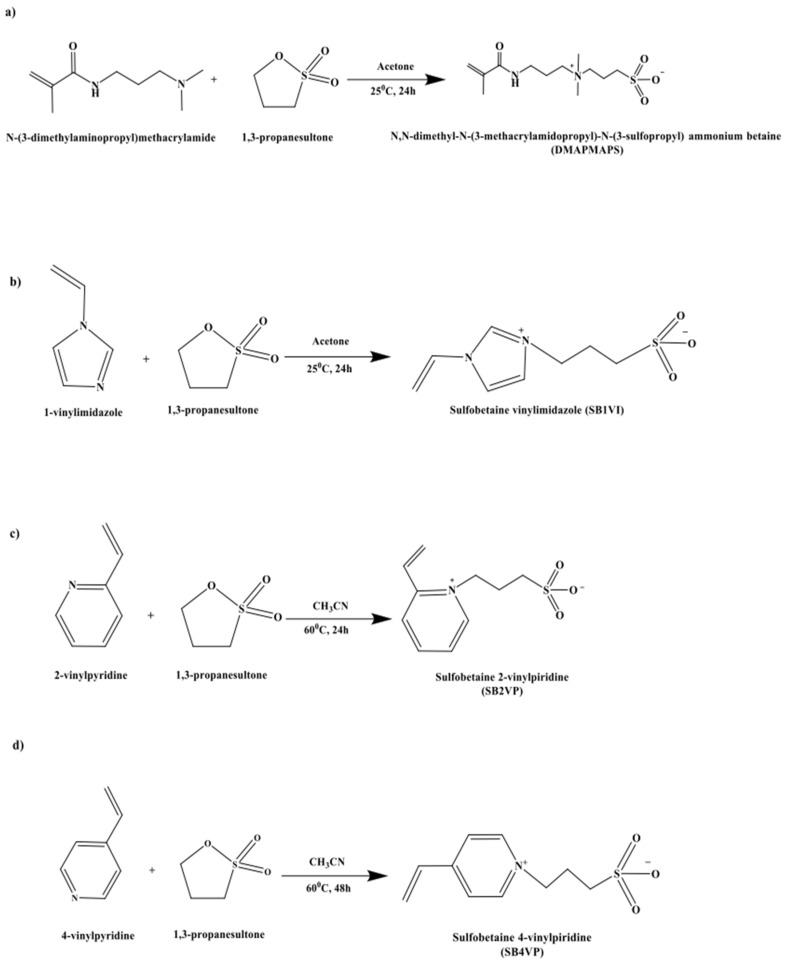
Reaction Schemes for the Synthesis of Monomers via Nucleophilic Ring Opening of 1,3-PS by Various Tertiary Amines: (**a**) DMAPMAPS, (**b**) SB1VI, (**c**) SB2VP, and (**d**) SB4VP.

*N*,*N-*dimethyl*-N*-(3-methacrylamidopropyl)-*N*-(3-sulfopropyl) ammonium betaine *N*,*N*-dimethyl-*N*-(3-methacrylamidopropyl)-*N*-(3-sulfopropyl) ammonium betaine (DMAPMAPS) was synthesized by reacting *N*,*N-*dimethylaminopropyl methacrylamide (DMAPMA) with 1,3-PS, as illustrated in Scheme 1a. Specifically, 8.51 g of DMAPMA was dissolved in 19.13 mL of acetone in a three-necked flask. This solution was then slowly introduced to a mixture of 4.71 mL of 1,3-PS in 19.13 mL of acetone over the course of 0.5 h under continuous stirring. The reaction mixture was stirred for 24 h at room temperature, resulting in the formation of a white, powdery product. This product was subsequently filtered, washed with acetone, and dried at 40 °C under reduced pressure in a vacuum oven for 24 h. The monomer was synthesized with a yield of ~70.9%.

##### Synthesis of Sulfobetaine 1-Vinylimidazole

Sulfobetaine 1-vinylimidazole (SB1VI) was synthesized by reacting vinylimidazole with 1,3-PS, as shown in Scheme 1b. In a three-necked flask, 5.00 mL (1 mol) of vinylimidazole was dissolved in 19.13 mL of acetone and stirred for 0.5 h. A solution of 4.77 mL of 1,3-PS in 19.13 mL of acetone was then added dropwise over a period of 0.5 h. The reaction mixture was stirred at ambient temperature for 24 h. The resulting white powder was filtered, washed with acetone, and dried at 40 °C under reduced pressure in a vacuum oven for 24 h. The monomer was synthesized with a yield of ~85.3%.

##### Synthesis of Sulfobetaine 2-Vinylpyridine

SB2VP monomer was synthesized through the quaternization of 2-vinylpyridine with 1,3-PS, as depicted in Scheme 1c. In a round-bottom flask, 5 mL of 2-vinylpyridine and 4.76 mL of 1,3-PS were dissolved in 50 mL of acetonitrile. The flask was sealed with a rubber membrane, purged with nitrogen gas for 20 min, and stirred in an oil bath at 60 °C. After 24 h, a pale-yellow solid precipitated. The product was filtered under vacuum, stirred in diethyl ether twice for at least eight hours each time, and dried at 40 °C under reduced pressure in a vacuum oven for 24 h. The monomer was synthesized with a yield of ~79%.

##### Synthesis of Sulfobetaine 4-Vinylpyridine

SB4VP monomer was synthesized using the quaternization reaction between 4-vinylpyridine and 1,3-PS, as illustrated in Scheme 1d. The reaction was conducted for 48 h to ensure complete conversion. The monomer was synthesized with a yield of ~82%.

#### 2.2.2. Preparation of Zwitterionic Polymers

##### Polymerization of DMAPMAPS

**Scheme 2:**
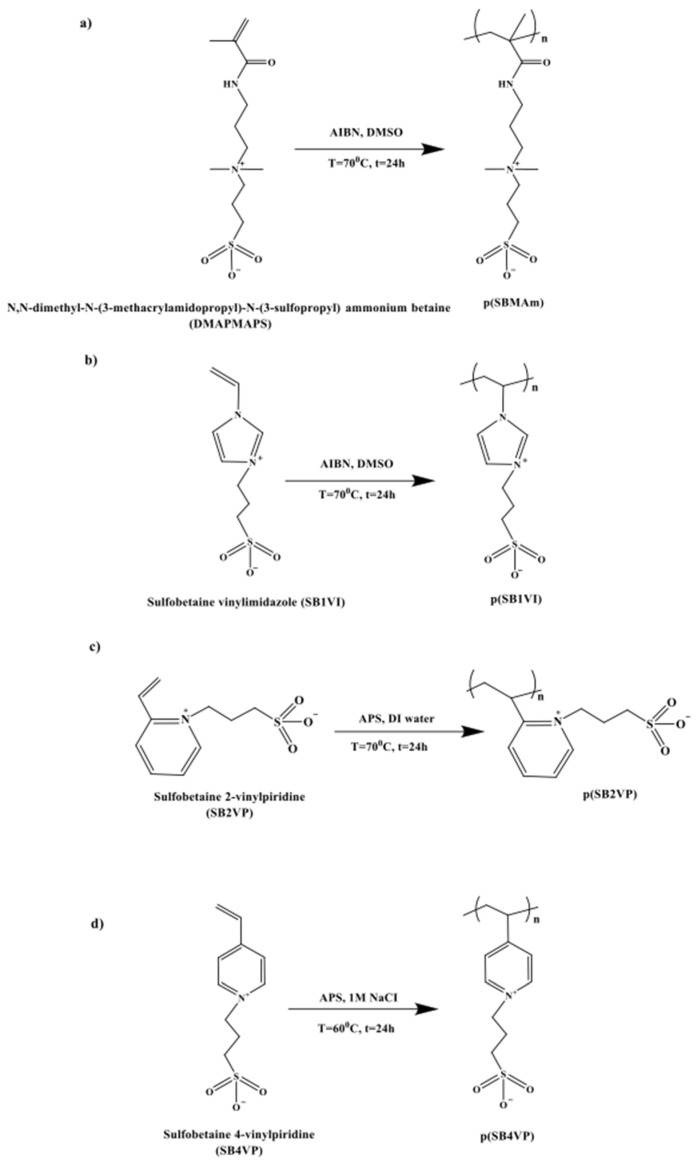
Polymerization Schemes for the Synthesis of Polymers: (**a**) pSBMAm (**b**) pSB1VI; (**c**) pSB2VP; (**d**) pSB4VP.

The polymerization of DMAPMAPS was conducted via free radical polymerization (FRP). Initially, 3.0 g of DMAPMAPS was dissolved in 60 mL of DMSO in a round-bottom flask under continuous stirring. Following this, 0.01 g of azobisisobutyronitrile (AIBN) was introduced, and the flask was sealed with a rubber membrane. The solution was purged with nitrogen gas for 20 min before being placed in an oil bath at 70 °C with continuous stirring. The polymerization reaction proceeded for 24 h. Subsequently, the reaction was terminated by adding 0.15 g of MEHQ. The homopolymer was precipitated using a 50:50 ethanol/hexane mixture. The resulting product was subjected to vacuum filtration, and residual solvent and monomers were extracted by washing the polymer twice with methanol, each wash lasting a minimum of 8 h. The final product was dried overnight in a vacuum oven at 40 °C (Scheme 2a). The final yield was ~62.1%.

##### Polymerization of SB1VI

The polymerization of sulfobetaine vinylimidazole (SB1VI) followed a similar procedure. The 3.0 g of SB1VI were dissolved in 60 mL of DMSO in a round-bottom flask with continuous stirring. AIBN (0.01 g) was added, and the flask was sealed and purged with nitrogen gas for 20 min. The polymerization reaction was carried out at 70 °C for 24 h. MEHQ (0.15 g) was introduced to quench the reaction. The resulting polymer was precipitated using a 50:50 ethanol/hexane mixture, vacuum filtered, and washed twice with 50:50 ethanol/hexane mixture, with each wash lasting a minimum of 8 h. The final product was dried overnight in a vacuum oven at 40 °C (Scheme 2b). The final yield was ~68%.

##### Polymerization of SB2VP

The synthesis of poly(SB2VP) was conducted via free radical polymerization. The 3.0 g of SB2VP were dissolved in 60 mL of DI water in a round-bottom flask under continuous stirring. APS (0.1 g) was added, and the flask was sealed and purged with nitrogen gas for 20 min. The sealed flask was then placed in an oil bath at 70 °C with stirring for 24 h. MEHQ (0.15 g) was introduced to quench the reaction. The resulting polymer was precipitated in ethanol, followed by vacuum filtration. Residual solvents and monomers were removed by washing the polymer twice with ethanol, with each wash lasting a minimum of 8 h. The final product was dried overnight in a vacuum oven at 40 °C (Scheme 2c) [36]. The final yield was ~59%.

##### Polymerization of SB4VP

The synthesis of poly(SB4VP) commenced by dissolving 3.0 g of SB4VP monomer in 60 mL of 1 M NaCI solution in a round-bottom flask under stirring. The initiator, APS (0.06 g), was dissolved in 1 mL of 1 M NaCI and added to the solution. The flask was tightly sealed with a rubber stopper and purged with nitrogen gas for 20 min. The polymerization was carried out in an oil bath at 60 °C for 24 h. The polymer was precipitated using a mixture of acetone/methanol (50:50 volume ratio). The resulting polymer was then dried in a vacuum oven at 40 °C for 24 h (Scheme 2d) [36]. The final yield was ~67%.

## 3. Polymer Characterization

### 3.1. NMR Characterization

A JNM-ECA 500 MHz NMR spectrometer (Boston, BOS, MA, USA) was used to characterize zwitterionic monomers and polymers. For 1H NMR spectra, a solution of zwitterionic monomers DMAPMAPS and SB1VI was prepared in (0.5% in deuterated water) D_2_O and in SB4VP 1 M NaCl in (0.5% in deuterated water) D_2_O. Additionally, the polymers pSBMAm and pSB2VP were also prepared in 0.5% deuterated water (D_2_O), whereas pSB1VI and pSB4VP were prepared in 1 M NaCl in 0.5% deuterated water (D_2_O).

### 3.2. FTIR Measurement

The chemical makeup of zwitterionic monomers and polymers was analyzed utilizing a Nicolet iS10 FTIR Spectrometer (Waltham, WLM, MA, USA), a Fourier transform infrared (FTIR) instrument.

### 3.3. CHNS Measurement

Elemental analysis of the polymers was recorded on a CHNS-O UNICUBE instrument (Langenselbold, LS, Germany). The initial mass of the sample was ~5 mg.

### 3.4. XRD Measurement

The SmartLab X-ray Diffraction (XRD) System by Rigaku (Tokyo, TYO, Japan), with a measuring range of Thetas/Thetad −3° to 160° 2 Theta, was utilized for phase identification of the specimens.

### 3.5. Characterization of Self-Assembled Morphology

The self-assembled morphology of the synthesized zwitterionic polymers was characterized using TEM and zeta potential measurement under various pH conditions. A JEOL JEM-1400 Plus transmission electron microscope (Boston, BOS, MA, USA) and a Malvern Zetasizer Nano series (Worcestershire, Worcs, UK) were employed for this analysis. Solutions of pSBMAm and pSB2VP were prepared in deionized water at a concentration of 1 mg/mL at 25 °C, while pSB4VP and pSB1VI were prepared in 1 M NaCl at the same concentration and temperature. These solutions were prepared for zeta potential measurements, which were taken in the range of ±30 mV.

### 3.6. Thermal Stability of Synthesized Sulfobetaine

TGA of the polymers was recorded using an STA 6000 (Shelton, CT, USA). The initial sample weight was ~40 mg, the heating rate was set to 10 °C/min, and the analysis was carried out in the range of 15–800 °C in a nitrogen atmosphere.

### 3.7. Characterization of Molecular Weight

Gel permeable chromatography (GPC) was carried out on a Viscotek GPC/SEC ((Worcestershire, Worcs, UK); Malvern Panalytical’s OMNISEC 5.02 multi-detector (Worcestershire, Worcs, UK) and Software was used. For the GPC measurements of water-soluble polymers, a 0.1 M KNO_3_ solution served as the solvent. The flow rate was set at 1 mL/min, with a column temperature of 30 °C and a detector temperature of 25 °C. Polyethylene oxide (PEO) served as a reference for the calibration curve establishment. The volume of the injected sample was 100 μL.

## 4. Results and Discussion

### 4.1. Monomer and Polymer Characterization

For the series of PZIs depicted in Figure 1, each monomer (DMAPMA, SB1VI, SB2VP, and SB4VP) was synthesized via the nucleophilic ring-opening of 1,3-PS with various vinyl aliphatic and aromatic tertiary amines. The synthetic procedures for the monomers are detailed in Scheme 1. The resulting monomers were obtained as white to yellowish-white powders, which were subjected to triple purification using diethyl ether to ensure high purity.

The chemical integrity and structure of the synthesized monomers were confirmed through comprehensive spectroscopic analyses, specifically ^1^H NMR and FTIR spectroscopy. The ^1^H NMR spectra provided detailed information on the chemical environments of the hydrogen atoms in the monomers, while the FTIR spectra confirmed the presence of the characteristic functional groups, and all this is illustrated in Table 1.

The FTIR and ^1^H NMR analyses of SBMAm, SB1VI, SB2VP, and SB4VP exhibited characteristic peaks indicative of their chemical functionalities.

**Figure 2:**
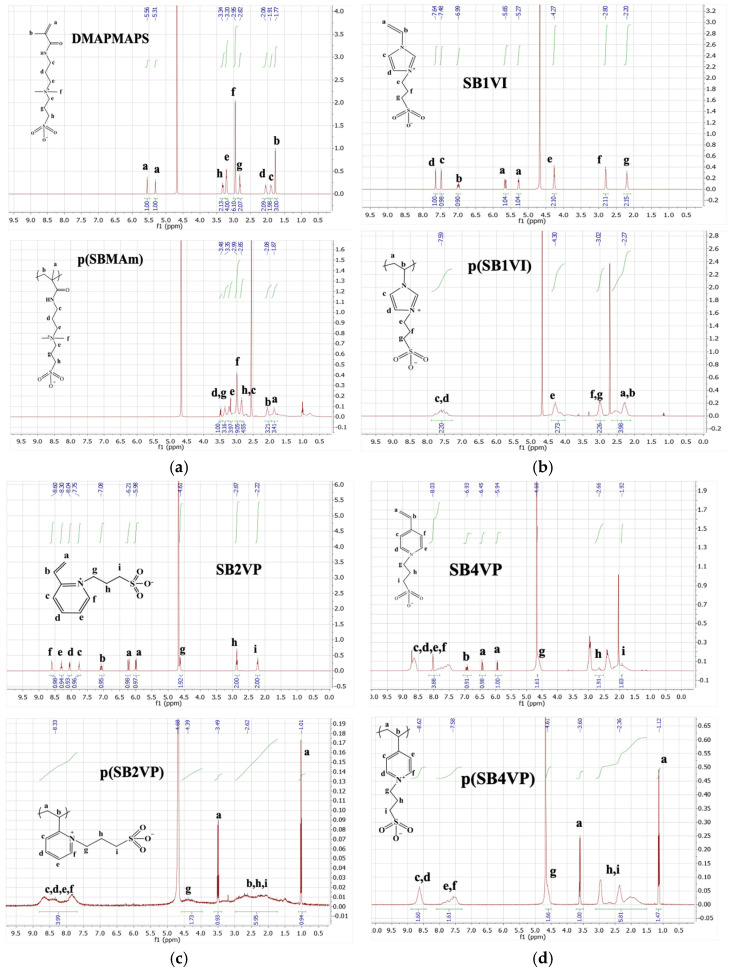
^1^H NMR Spectra of Polyzwitterionic Polymers. (**a**) pSBMAm, (**b**) pSB1VI, (**c**) pSB2VP, and (**d**) pSB4VP.

The ^1^H NMR spectra of the pSBMAm polymer in D_2_O exhibited distinct peaks corresponding to internal aliphatic methylene protons in the range of 1.8–3.4 ppm. Examination of the ^1^H NMR spectrum of the SBMAm monomer indicated aliphatic proton signals attributed to C=C stretching vibrations at approximately 5.56 and 5.31 ppm, which experienced a shift to the region of 3.41 ppm after polymerization (Figure 2a).

**Figure 3:**
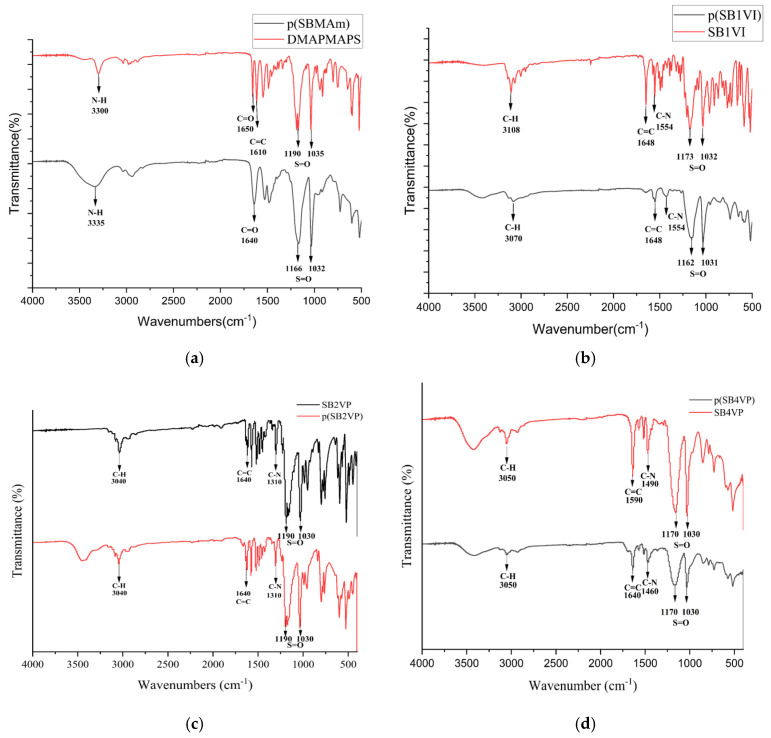
FTIR Analysis of Synthesized Polymers. (**a**) pSBMAm, (**b**) pSB1VI, (**c**) pSB2VP, and (**d**) pSB4VP.

In sulfobetaine methacrylamide, distinctive peaks at 1610 cm^−1^ (C=C stretching vibration), 1190 and 1035 cm^−1^ (S=O moieties), and 3300 cm^−1^ (N-H stretching vibrations) denoted its key structural components. Conversely, the polymerized form, pSBMAm, showcased notable changes, with peaks at 1640 cm^−1^ (C=O stretching vibration), 3335 cm^−1^ (N-H stretching vibrations), and 1166 and 1032 cm^−1^ confirming S=O functionalities, indicative of the polymerization process-induced alterations (Figure 3a).

The ^1^H NMR spectra of the pSB1VI in D_2_O displayed prominent peaks associated with internal aromatic protons within the range of 7.64–7.48 ppm. Analysis of the ^1^H NMR spectrum of the SB1VI monomer revealed aliphatic proton signals related to C=C stretching vibrations at around 5.22 and 5.56 ppm, which subsequently shifted to the region of 3.98 ppm after polymerization (Figure 2b).

Given the pronounced quadrupolar effects of ^14^N, the absence of or significant reduction in signals for protons in the -CH= group of the SB1VI monomer and p(SB1VI) polymer can be attributed to the influence of nitrogen’s quadrupole moment. Roberts (1958) [23] observed that interactions involving ^14^N frequently lead to signal broadening or even complete loss, as nitrogen’s quadrupole moment induces rapid relaxation and reduces signal visibility.

In addition, the hydrogen signal in the -NH group of the DMAPMAPS monomer and its polymer, p(SBMAm), may also disappear or weaken in the NMR spectrum due to quadrupolar interactions with ^14^N. Nitrogen-14’s quadrupole moment causes faster relaxation, which can blur proton signals in close proximity, including those of -NH groups. In systems like ours, ^14^N can “flip” its quantum orientation states, disrupting stable proton resonance and leading to considerable broadening or even complete signal loss. This effect complicates the analysis of proton spectra in nitrogen-containing aromatic compounds, as is evident in our study [23].

Similarly, for SB1VI, prominent peaks at 1648 cm^−1^ (C=C stretching vibration), 1173 and 1032 cm^−1^ (S=O moieties), and 1554 cm^−1^ (C-N bonding) characterized its molecular structure. In contrast, the polymer form, pSB1VI, exhibited persistent peaks at 1648 cm^−1^ (C=C stretching vibration), 1648 cm^−1^ (C-N bonding), and 1162 and 1031 cm^−1^, indicating retained S=O functionalities and suggesting structural stability after polymerization (Figure 3b).

The ^1^H NMR spectra of pSB2VP and pSB4VP polymers containing pendant sulfobetaine moieties were acquired in D_2_O [36]. In the ^1^H NMR spectrum, the characteristic peaks corresponding to aromatic protons of the original pSB2VP and pSB4VP polymers were identified at 8.33 ppm and 7.58–8.62 ppm. The internal aliphatic methylene proton peaks were discernible at 2.22 and 2.88–4.63 ppm. Analysis of the ^1^H NMR spectrum of SB2VP and SB4VP monomers revealed aliphatic proton peaks associated with C=C stretching vibrations in the range of 5.98 and 6.21–7.08 ppm, which subsequently shifted to the range of 1.01–2.62 ppm following polymer synthesis (Figure 2b).

Moreover, for SB2VP, crucial peaks at 1640 cm^−1^ (C=C stretching vibration), 1310 cm^−1^ (C-N bonding), and 1190 and 1030 cm^−1^ (S=O moieties) defined its chemical composition. In its polymer form, pSB2VP, consistent peaks at 1640 cm^−1^ (C=C stretching vibration), alongside peaks at 1187 and 1030 cm^−1^ confirming S=O functionalities, indicated the maintenance of essential structural components post polymerization (Figure 3c).

Lastly, in SB4VP, vital peaks at 1590 cm^−1^ (C=C stretching vibration), 1400 cm^−1^ (C-N bonding), and 1170 and 1030 cm^−1^ (S=O moieties) characterized its molecular structure. Its polymerized counterpart, pSB4VP, displayed distinct peaks at 1640 cm^−1^ (C=C stretching vibration) and 1168 and 1030 cm^−1^ (S=O functionalities), with an emergent peak at 1460 cm^−1^ (C-N bonding) (Figure 2d) indicating alterations due to the polymerization process. These comprehensive spectral findings elucidate the distinctive chemical compositions and structural transformations induced by polymerization across the sulfobetaine derivatives.

The CHNS elemental analysis supports the successful synthesis of the zwitterionic polymers, indicating only minor deviations between experimental and theoretical values, as presented in Table 2. For pSBMAm and pSB1VI, slight variations in carbon content suggest subtle structural differences within the polymer chains, while sulfur, nitrogen, and hydrogen values show close alignment with calculated values, affirming the accuracy of the synthetic process

For the regio-isomers pSB2VP and pSB4VP, sulfur and nitrogen values remained consistent with theoretical expectations. However, a recurring, modest reduction in carbon content relative to theoretical values may reflect slight heterogeneity in polymer structure, which can influence elemental composition.

Moreover, CHNS analysis, particularly in complex polymers, can present inherent challenges. Subtle deviations in carbon values may arise from small variations in backbone structure or the possible inclusion/exclusion of low-molecular-weight fragments, which can lead to minor differences when compared to idealized theoretical models.

Overall, the CHNS analysis verified the intended chemical structures of the synthesized polymers, with minor deviations likely due to structural variations during polymerization. These findings underscore the success of the polymer synthesis and provide valuable insights to enhance polymerization conditions for future consistency in material properties.

### 4.2. Structural Characterization

**Figure 4:**
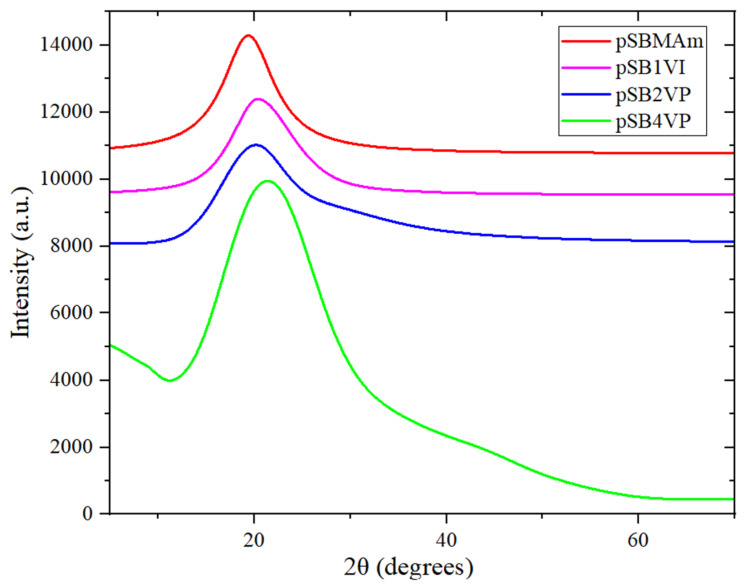
Wide-angle X-ray patterns for the different polymer systems.

**Figure 5:**
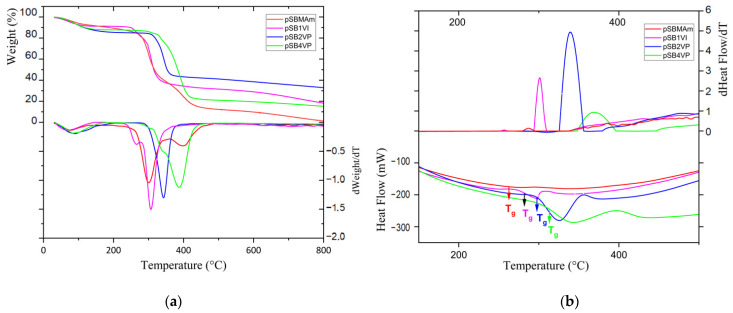
TGA and DSC of Polyzwitterionic Polymers. (**a**) TGA; (**b**) DSC.

The XRD patterns (Figure 4) of the zwitterionic polymers pSBMAm, pSB1VI, pSB2VP, and pSB4VP display large peaks between 10° and 30° 2θ, indicating an amorphous structure due to lack of long-range order between polymer chains. Zwitterionic groups with both cationic and anionic functions produce repulsive forces and strong electrostatic contacts, preventing crystalline domain formation and disrupting chain packing. The XRD patterns show diffuse scattering, which is typical of amorphous materials, while the big peaks indicate short-range order inside the matrix. Hydration of zwitterionic groups further inhibits crystallization. DSC curves also show the absence of a melting peak, which supports the amorphous state of polymers (Figure 5b).

This amorphous nature is indicative of potential glass-forming ability, a characteristic that is significant for materials designed for specific thermal and mechanical applications. The absence of crystalline order not only affects the thermal properties but also provides an opportunity to tailor the polymer’s behavior through precise chemical modifications. The glass-forming ability of these zwitterionic polymers will be further analyzed in subsequent sections, highlighting its implications for advanced material design.

### 4.3. Thermal Analysis

Polyzwitterions with linear and imidazole/pyridine ring structures can significantly affect TGA and DSC analysis by increasing their temperature stability. Linear structures allow for easier rotation of the polymer chains, whereas the introduction of rings, especially when increasing from five- to six-membered rings, restricts this rotation, thereby altering the thermal properties. Additionally, the ortho and para configurations of the substituent groups on the aromatic rings can further influence these properties. By manipulating the chemical structure, such as the degree of ring incorporation and the position of substituent groups, we can effectively control the thermal stability and properties of these polymers.

These polymers tend to absorb water from their surroundings due to their hygroscopic nature. To eliminate loosely bound water and prevent re-absorption, the cast PZI powders were kept in a vacuum oven at 25 °C. Accurate and precise measurement of the specific heat capacity of polyzwitterions relies on knowing their mass without any bound water. To determine the exact mass of the PZIs, it is necessary to measure the amount of absorbed water. TGA was utilized to assess both the absorbed water content and the thermal degradation temperature of the PZIs.

The percent of mass remaining for PZIs as a function of temperature from 30 to 800 °C showed distinct stages of mass loss (Figure 5a). An initial mass-loss step began at 30 °C and concluded around 200 °C, attributing to the evaporation of absorbed water and other volatile components. This was followed by a significant mass loss starting around 300 °C due to the thermal degradation of the polymer. The mass remaining after degradation plateaued at different temperatures for each type of PZI, with degradation being largely completed by 450 °C.

As the temperature increased to 800 °C, the remaining mass did not drop to zero. The PZIs retained between 20% and 40% of their mass due to degraded polymer char that adhered to the bottom of the TGA basket and was not removed under nitrogen purge. The methacrylamide-based polyzwitterion exhibited less char residue, indicating a more complete degradation compared to the PZIs with aromatic side groups (pSB1VI, pSB2VP, and pSB4VP). This observation suggests that the presence of aromatic rings in the polymer structure contributes to the formation of residual char.

A magnified view of the first mass loss step reveals that it was caused by the loss of bound water. The mass of bound water is determined by finding the height of the step change between 30 °C and 200 °C. The amount of bound water varies from approximately 8% to 9% for the polyzwitterion without an aromatic structure (pSBMAm) and varies from approximately 10% to 15% for the polyzwitterions containing an aromatic ring.

The methacrylamide backbone of pSBMAm is more hydrophilic compared to the backbones of pSB1VI, pSB2VP, and pSB4VP, which contain aromatic rings. This increased hydrophilicity explains the higher water absorption observed in pSBMAm, as it more readily interacts with and retains water molecules from the environment. In contrast, the presence of aromatic and imidazole rings in pSB1VI, pSB2VP, and pSB4VP imparts a more hydrophobic character, reducing their affinity for water absorption.

Bound water is fully removed in all the polyzwitterions around 200 °C, and there is no mass lost to degradation until the temperature increases above 250 °C, as seen in Table 3. pSBMAm shows the lowest thermal stability with a degradation onset at 301.03 °C, while the other three PZIs show degradation-onset temperatures 30–80 °C higher. pSB4VP has the highest decomposition temperature (Td), indicating its superior thermal stability, likely due to the para-substitution, which reduces spatial interference and enhances its thermal properties. This tendency shows that the improved thermal stability of pSB4VP is likely linked to its lower steric hindrance compared to ortho-substitution in pSB2VP, allowing for more effective π–π stacking interactions among its pyridine rings. This trend in Td reflects the increasing thermal stability conferred by the imidazole and pyridine rings present in the polymer structures. All four PZIs exhibit a multi-step degradation profile, a characteristic observed in other zwitterionic systems [40].

DSC was employed to study the thermal transitions, specifically the Tg, of the polymers (Figure 5b): pSBMAm, pSB1VI, pSB2VP, and pSB4VP. The Tg is a crucial parameter that indicates the temperature at which the polymer transitions from a hard, glassy material to a soft, rubbery state.

The DSC analysis revealed distinct Tg for each polymer, reflecting the influence of their chemical structure on thermal behavior. As seen in Table 3, pSBMAm exhibited the lowest Tg, which can be attributed to its hydrophilic methacrylamide backbone. This flexible backbone allows for greater molecular mobility, resulting in a lower Tg. In contrast, the presence of aromatic rings in pSB1VI, pSB2VP, and pSB4VP contributes to higher Tg values. The rigid nature of these rings restricts molecular mobility, leading to higher Tg compared to pSBMAm [41].

These findings from DSC are consistent with the observations from TGA. The TGA results showed that pSBMAm had the lowest Td, indicating lower thermal stability compared to the other PZIs. This lower thermal stability correlates with the lower Tg observed for pSBMAm. Conversely, the higher Td of pSB1VI, pSB2VP, and pSB4VP observed in TGA align with their higher Tg values from DSC, suggesting a direct relationship between the rigidity imparted by the aromatic and imidazole rings and the overall thermal stability of the polymers.

The distinct multi-step degradation profiles observed in TGA for these polyzwitterions can also be related to their Tg values. The polymers with higher Tg values exhibited more stable thermal behavior, as indicated by their higher Td and more complete degradation profiles. This relationship underscores the significant impact of chemical structure on both the glass transition and thermal degradation behaviors of PZIs.

The Tg have several implications for the applications and performance of these PZIs. Polymers with higher Tg values, such as pSB1VI, pSB2VP, and pSB4VP, are expected to maintain their mechanical integrity at higher temperatures compared to pSBMAm. Higher Tg values generally correlate with greater stiffness and strength in the polymer’s glassy state, suggesting that pSB1VI, pSB2VP, and pSB4VP may exhibit superior mechanical properties in applications requiring structural stability [42].

These findings highlight the critical role of chemical structure in determining the thermal and mechanical properties of polyzwitterionic polymers, guiding their design and application in various fields requiring specific thermal stability and performance characteristics.

### 4.4. Self-Assembly and Molecular Weight Analysis

Zeta potential plays a crucial role and is related to the ionization state of zwitterionic groups, as demonstrated by the self-assembly behavior of polyzwitterionic polymers, namely pSBMAm and pSB2VP, under varying pH conditions. The polymer’s net charge, which directly affects the zeta potential and, in turn, the polymers’ self-assembly behavior, is determined by the balance between the positive and negative charges inside these groups.

**Figure 6:**
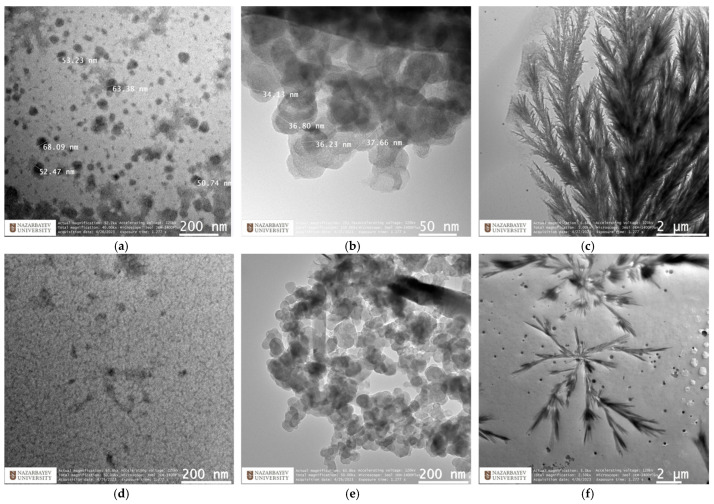
TEM images of polymers pSBMAm and pSB2VP. (**a**,**d**) pH = 4, (**b**,**e**) pH = 8, and (**c**,**f**) pH = 12.

**Figure 7:**
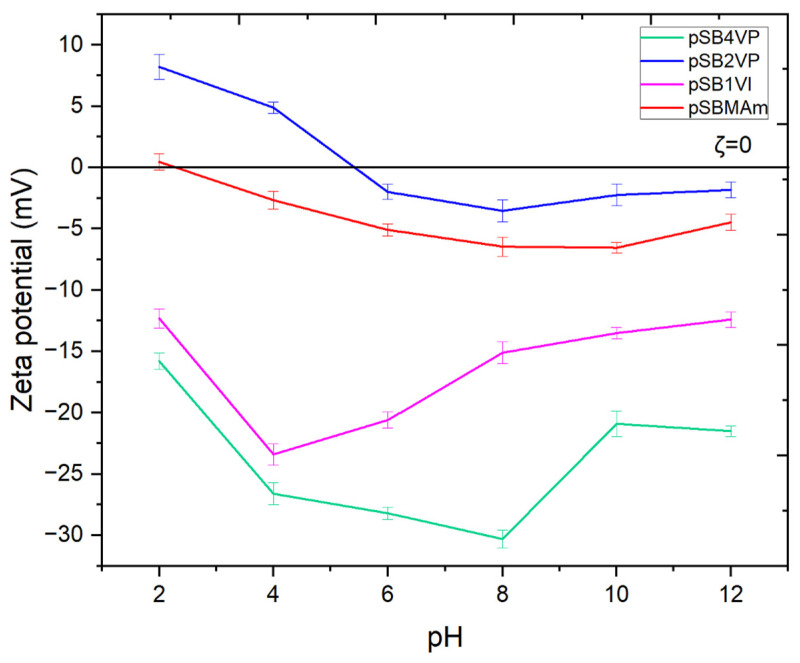
Zeta Potential Measurements of PZIs at Various pH Levels.

The selection of pSBMAm and pSB2VP for TEM analysis was based on their excellent solubility in water and notable pH sensitivity [42], as demonstrated by the zeta potential measurements shown in Figure 7. In contrast, the limited solubility of pSB1VI and pSB4VP in water and their dependence on saltwater conditions posed significant challenges for zeta potential measurements and rendered the TEM analysis (Figure 6) of these two polymers impractical.

Polymer chains experience the least amount of electrostatic repulsion at pH values close to the isoelectric point, where the polymer’s net charge is almost zero. Small, stable micelles are easier to develop under these conditions. For example, at pH 4, pSBMAm (pH 2.3) generates spherical micelles with dimensions ranging from 20 to 50 nm. The low electrostatic forces at pH 6 cause these structures to stay mostly unaltered. Analogously, at pH 4 and 8, pSB2VP, which has an isoelectric point of 5.45, forms micelles that are between 30 and 40 nm in size.

The balance between the zwitterionic charges is upset as the pH moves away from the isoelectric point, changing the zeta potential. Increased protonation causes the polymer to tend to gain a net positive charge at lower pH values, whereas deprotonation causes a net negative charge at higher pH levels. Larger, more loosely coupled aggregates are formed as a result of these modifications, which also increase electrostatic repulsion between polymer chains. The formation of bigger spherical structures, such as fractal or dendritic aggregates, was observed in both pSBMAm and pSB2VP at pH = 12, where a notable swelling was observed. These morphological alterations are mostly caused by an increase in electrostatic repulsion at higher pH values. The TEM images and ζ potential measurements demonstrate that both pSBMAm and pSB2VP exhibit pH-sensitive self-assembly behavior, highlighting the critical role of chemical structure and electrostatic interactions in the self-assembly of polyzwitterionic polymers and providing insights into their potential applications in fields requiring precise control over polymer morphology and stability.

**Figure 8:**
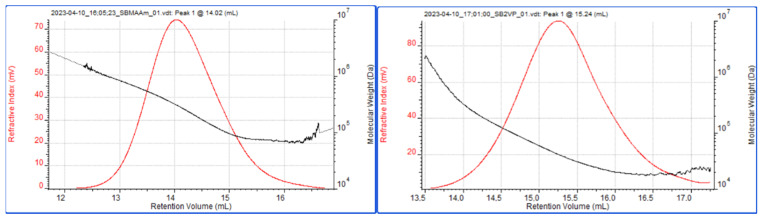
GPC analysis results for pSBMAm and pSB2VP.

GPC analysis was conducted to determine the molecular weight distribution of two synthesized PZIs: pSBMAm and pSB2VP, shown in Figure 8. The obtained data, including Mn, Mw, Mz, Mp, and PĐI, are shown in Table 4.

The GPC analysis revealed significant variations in molecular weight distribution and polydispersity between pSBMAm and pSB2VP. The PĐI values for pSBMAm and pSB2VP are 1.541 and 1.698, respectively, indicating a broader molecular weight distribution for pSB2VP compared to pSBMAm. Additionally, pSBMAm exhibited significantly higher Mn and Mw values compared to pSB2VP, suggesting that pSBMAm has a larger average molecular weight. The lower molecular weights of pSB2VP can be attributed to its specific chemical structure, which might limit chain growth during polymerization.

The differences in molecular weight distribution can be attributed to the distinct chemical structures of the polymers. The methacrylamide group connecting the side group to the backbone in pSBMAm results in a more flexible polymer chain, leading to higher molecular weights. In contrast, the presence of the pyridine ring in pSB2VP introduces rigidity to the polymer chain, resulting in lower molecular weights and a broader distribution due to possible limitations in chain mobility and growth.

TEM analysis further elucidated the self-assembly behavior of these polymers. At pH 12, TEM images show that pSBMAm forms larger fractal structures compared to pSB2VP. While the larger size observed in the TEM images suggests differences in structural organization, it does not conclusively indicate a greater propensity for self-assembly. Quantitative metrics, such as the critical micelle concentration (CMC), would be required for a definitive assessment and represent a key direction for future research.

These GPC and TEM results have several implications for the thermal and mechanical properties of the polymers. Higher molecular weights generally enhance the thermal stability of polymers. Therefore, pSBMAm is expected to exhibit better thermal stability compared to pSB2VP. Additionally, polymers with higher molecular weights and narrower distributions often demonstrate improved mechanical strength. Hence, pSBMAm may possess superior mechanical properties, making it suitable for applications requiring high strength and durability.

These insights are crucial for understanding the relationship between the chemical structure of polyzwitterions and their resulting properties, guiding the design and application of these materials in various fields.

## 5. Conclusions

This study comprehensively investigated the thermal behavior, molecular weight, and self-assembly properties of a series of polyzwitterionic polymers, including pSBMAm, pSB1VI, pSB2VP, and pSB4VP. Thermogravimetric analysis and differential scanning calorimetry revealed distinct glass transition temperatures for these polymers, underscoring the significant influence of their chemical structures. The data indicated that pSBMAm exhibits the lowest Tg, attributable to its hydrophilic methacrylamide backbone, whereas the incorporation of aromatic and imidazole rings in pSB1VI, pSB2VP, and pSB4VP results in higher Tg values, demonstrating the pivotal role of chemical structure in modulating the thermal properties of polyzwitterions. TGA and DSC revealed distinct glass transition temperatures of 276.52 °C for pSBMAm, 280.11 °C for pSB1VI, 297.04 °C for pSB2VP, and 313.69 °C for pSB4VP. Gel permeation chromatography analysis highlighted that pSBMAm possesses a higher molecular weight (Mn = 197,241 g/mol) relative to pSB2VP (Mn = 35,810 g/mol), indicating notable differences in polymerization behavior. Transmission electron microscopy observations at pH 12 further elucidated that pSBMAm forms larger fractal/dendritic structures compared to pSB2VP, suggesting a propensity for more extensive polymerization and self-assembly in pSBMAm.

The study revealed that side groups significantly impact the glass transition temperature and self-assembly behavior of these polymers. The functional groups present in the chemical structure influence both the thermal stability and self-assembly properties under varying conditions.

These findings underscore the paramount importance of chemical structure in determining the thermal and self-assembly properties of polyzwitterions. By strategically manipulating the polymer backbone and side groups, it is possible to precisely tailor the glass transition temperatures and self-assembly behaviors of these materials. This insight offers valuable opportunities for the design and synthesis of polymeric materials with the controlled stabilities essential for advanced applications. Understanding these behaviors provides a crucial foundation for developing materials that meet specific thermal property and stability requirements.

## Figures and Tables

**Table 1:** (FTIR) Data Analysis of Monomers and Polysulfobetaines.

Functional Groups (cm^−1^)/Polymers	SBMAm [36]	pSBMAm	SB1VI [37]	pSB1VI	SB2VP [38]	pSB2VP	SB4VP [39]	pSB4VP
C-H	-	-	3108	3070	3040	3040	3050	3050
C=C	1610	-	1648	1648	1640	1640	1590	1640
C-N	-	-	1554	1554	1310	1310	1490	1460
N-H	3300	3335	-	-	-	-	-	-
C=O	1650	1640	-	-	-	-	-	-
S=O	1190 1035	1166 1032	1173 1032	1162 1031	1190 1030	1190 1030	1170 1030	1170 1030

**Table 2:** Elemental Analysis of CHNS for the Different Zwitterionic Polymers.

Polymers	%N_E.A._ ^a^	%S_E.A._ ^a^	%H_E.A._ ^a^	%N_calc._ ^b^	%S_calc._ ^b^	%H_calc._ ^b^	%C_calc._ ^b^
pSBMAm	7.31	10.52	8.20	9.51	10.89	8.22	48.95
pSB1VI	10.54	15.29	6.35	12.95	14.83	5.59	44.02
pSB2VP	6.27	11.72	5.24	6.16	13.98	5.71	52.39
pSB4VP	5.36	12.15	6.59	6.16	13.98	5.71	52.39

^a^ %N and %S, %H, and %C determined by elemental analysis. ^b^ Stoichiometric calc %N and %S, %H, and %C calculated from according to the molar weight ratio of 14.01:32.06:1.00:12.01.

**Table 3:** Thermal Degradation and Glass Transition Data for Polyzwitterionic Polymers.

PZI	Stage	TGA Range (°C)	Mass Loss (%)	Evolved Moiety	T_d_ ± 0.5 (°C)	T_g_ ± 0.5 (°C)
pSBMAm	I	~30–180	8	Solvent	301.03	276.52
	II	~283–359	44	Polymer		
	III	~368–400	16	Zwitterionic group [38]		
pSB1VI	I	~30–200	11	Solvent	306.93	280.11
	II	~297–355	36	Polymer		
pSB2VP	I	~30–205	15	Solvent	342.53	297.04
	II	~315–361	36	Polymer		
pSB4VP	I	~30–200	13	Solvent	387.14	313.69
	II	~327–429	56	Polymer		

**Table 4:** GPC Results of Polyzwitterionic Polymers.

PZI\MW	Mn, Da	Mw	Mz	Mp	PĐI
pSBMAm	197.241	304.020	430.360	305.276	1.541
pSB2VP	35.810	60.822	206.875	41.394	1.698

## Data Availability

Data are contained within the article.
